# Research trends and hotspots of exercise for people with sarcopenic: A bibliometric analysis

**DOI:** 10.1097/MD.0000000000035148

**Published:** 2023-12-15

**Authors:** Wanli Zang, Haohao Chen, Jin Yan, Dong Li, Ningkun Xiao, Xiaoqin Zheng, Zezhong Zhang

**Affiliations:** a Postgraduate School, University of Harbin Sport, Harbin, China; b Centre for Active Living and Learning, University of Newcastle, Callaghan, NSW, Australia; c College of Human and Social Futures, University of Newcastle, Callaghan, NSW, Australia; d Department of International Culture Education, Chodang University, Jeollanam-do, Republic of Korea; e Department of Psychology, Ural Federal University, Yekaterinburg, Russia; f Department of Sports, Harbin University, Harbin, China.

**Keywords:** bibliometrics, exercise therapy, muscle mass, research hotspot, sarcopenia

## Abstract

This study aimed to analyze the trends and themes in exercise and sarcopenia research using a bibliometric approach. The Web of Science citation database was used to identify papers published on exercise and sarcopenia. The retrieved data on institutions, journals, countries, authors, journal distribution, and keywords were analyzed scientometric ally using CiteSpace and VOSviewer. 2895 papers were included according to our specified inclusion criteria eventually. The data showed an upward trend in the number of published articles on exercise and sarcopenia. The countries with the highest number of publications were the United States, Japan, and England; research institutions were mainly composed of universities in Europe and the United States, and high-producing authors formed major collaborative teams, but cross-geographical and cross-institutional collaboration was not apparent; research was closely focused on 3 aspects: resistance exercise, resistance combined with other forms of exercise, and exercise combined with nutritional supplementation, of which resistance exercise was a particular focus; and recently, the research hotspots were mainly the effects of exercise on grip strength. The most cited articles were consensus guidelines published by the working group on sarcopenia in the elderly from different continents. The prevention and rehabilitation of sarcopenia in the elderly are gaining attention. Current primary exercise therapies for sarcopenia and exercise combined with nutritional supplementation have significant advantages and the potential to delay muscle decay. This suggests a promising area for future research that could benefit from further advances.

## 1. Introduction

With advances in medical technology and improvements in social welfare, life expectancy among older adults has increased. However, an increase in life years does not always translate into a healthy lifestyle. Instead, it is often accompanied by disability, increased risk of chronic disease, and poor quality of life.^[1]^ Sarcopenia is a progressive and systemic skeletal muscle disease that usually occurs with age and is characterized by a decline in skeletal muscle strength and mass.^[2,3]^ It is associated with a range of adverse health outcomes^[4–6]^ and high social and economic costs.^[7]^ Currently, no drugs are approved for treating muscular osteoporosis, because muscular osteoporosis overall incidence ranges from 0.2% to 86.5%.^[2]^ Phase 2 clinical trials testing the effects of myostatin antibodies have shown a minimal impact on muscle function. Numerous studies have identified strategies to prevent and treat sarcopenia, focusing primarily on physical activity and nutrition.^[8]^ Exercise effectively prevents and treats muscle wasting syndrome, causing varying degrees of change in skeletal muscle mass and strength and improving balance.

Bibliometric analysis is a statistical method for exploring and analyzing scientific literature that can reveal global trends and research hotspots.^[9]^ It has been widely applied in information science, chemistry and physics, medicine, and many others, helping researchers, clinicians, and health policymakers collect information, understand specific research areas and their applications, and promote interdisciplinary collaborations. It helps researchers, clinicians, and healthcare policymakers gather information to understand particular research areas and their applications, and facilitates multidisciplinary collaboration.^[10]^ Given the considerable superiority of this approach, the application of bibliometrics is of great importance in exercise and sarcopenia research.

This study conducted a bibliometric analysis of exercise and sarcopenia research based on relevant papers published in Web of Science 2013 to 2022 to gain insight into the current status of exercise therapy for sarcopenia and to explore exercise promotion programs for sarcopenia to provide reference for future researchers.

## 2. Methods

### 2.1. Search strategy

The Web of Science Core Collection database, published by Clarivate, was used as the data source. The search was conducted by topic, and the search strategy was TS = (Aerobic OR Physical Activity OR Physical Exercise OR Exercise OR Resistance OR Combined Exercise OR Combined training OR Yoga OR Progressive resistance training OR Tai Chi OR Ba Duan Jin OR Qigong OR High-Intensity Interval Training) AND TS = (sarcopenia). The search data were collected on December 31, 2022, to avoid bias in the search results due to daily updates. 2012-12-31 to 2022-12-31 was selected as the time range, and the article type was the article, the language English, and the final data were 2895 records. Finally, the plain text file was exported. The exported data included the year of publication, title, author name, nationality, abstract, keywords, and journal name, which were exported to a txt file 6 times, and finally, the text was merged.

### 2.2. Research methodology

We used the CiteSpace V5.7.R1 (Drexel University, Chaomei Chen) to visualize and analyze the data. The merged text file was renamed as download_1.txt and saved in the input folder. The ormat converter that came with the CiteSpace 5.7.R1 was converted to a format suitable for CiteSpace and saved in an output folder. The time slice was set to one, the period was 2013 to 2022, and the time interval was one. A visual knowledge graph was created with the nodes of country/region, institution, author article, high-frequency keywords, and highly cited literature, and related data were obtained. The keywords were clustered using VOS viewer software version 1.6.18 (Leiden University, Leiden, The Netherlands) and distinguished by different colors to obtain the cluster map.^[11]^

## 3. Results

### 3.1. Analysis of the number of articles issued

**Figure 1.**
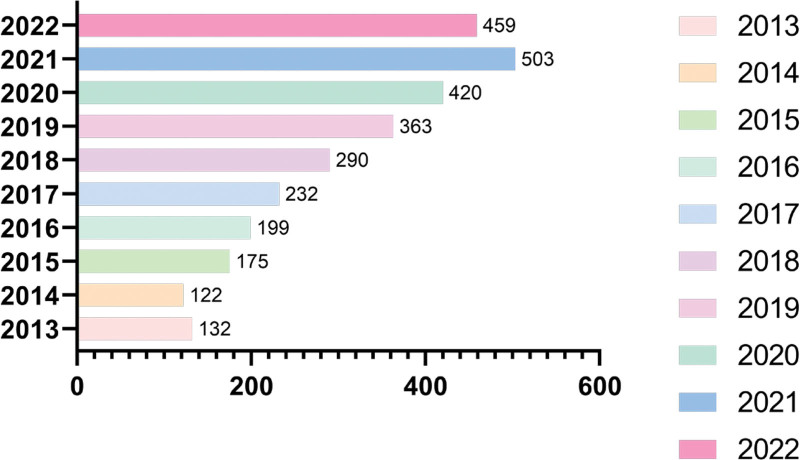
Distribution of related research publications from 2013 to 2022.

The international publication volume on exercise interventions for sarcopenia is shown in Figure 1, with 2895 articles published from 2013 to 2022, with an average annual volume of 289 articles. In terms of the overall trend, although the number of publications decreased slightly in some years, the number of publications is increased yearly. The highest number of publications reached 503 by 2021, which is generally the rising period.Research in this field is still receiving the attention of researchers.

### 3.2. Country analysis

**Figure 2.**
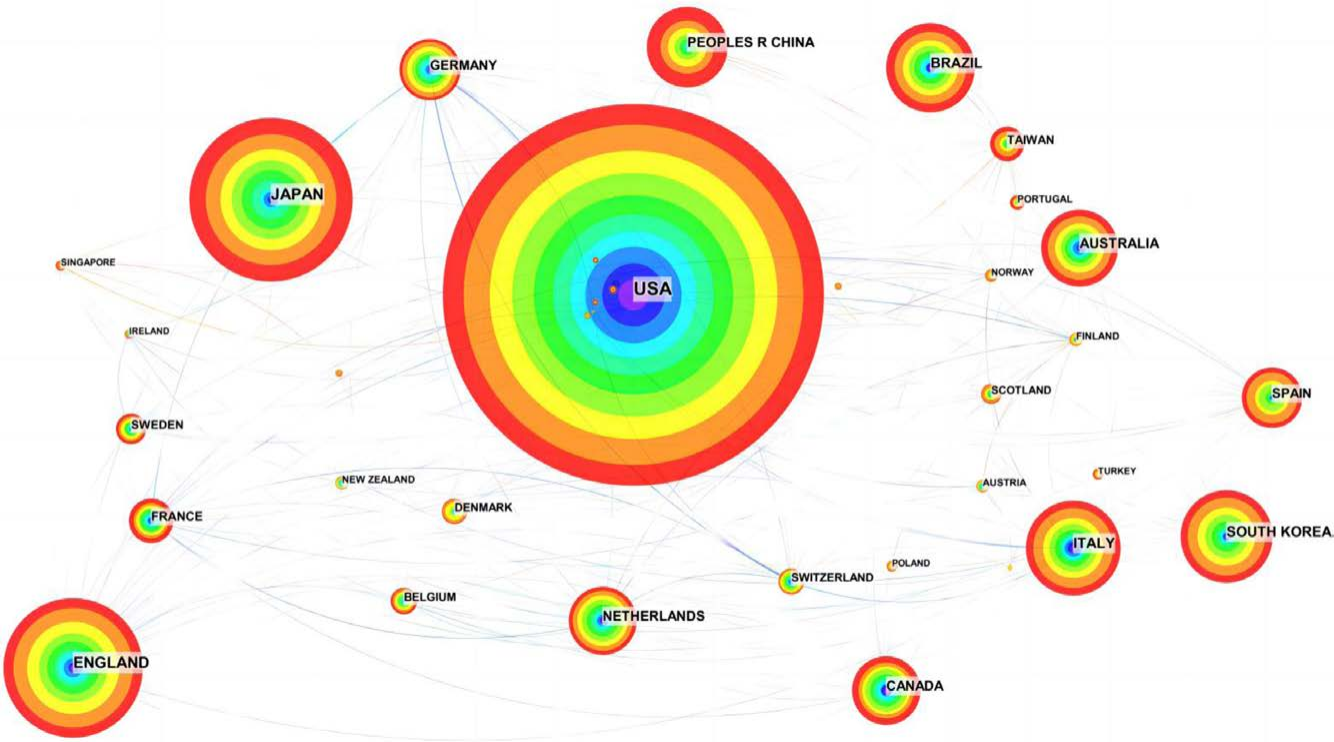
Distribution of countries (regions) publishing related literature, 2013 to 2022.

A country analysis can visually reveal the importance of each country in this direction. Figure 2 and Table 1 show the top ten countries in terms of the number of publications: the top 3 countries are the United States, Japan, and the United Kingdom. The United States (0.6) had the highest number of publications in this field, with 754% or 26.044%, followed by Japan (0,25) with 326% or 11.26%, the United Kingdom (0,24) with 279% or 9.637%, and China (0.08) with 164% or 5.664%, ranking seventh. The centrality of the top 5 countries was higher than 0.1, indicating that these countries play an important role in research in this field.

**Table 1:** Distribution of countries (regions) publishing relevant literature, 2013 to 2022.

	Count	Centrality	Country
1	754	0.6	USA
2	326	0.25	Japan
3	279	0.24	England
4	192	0.24	Italy
5	185	0.14	South Korea
6	180	0.17	Brazil
7	164	0.08	Peoples R China
8	157	0.22	Australia
9	141	0.18	Canada
10	140	0.14	Netherlands

### 3.3. Institutional analysis

Table 2 shows that the top ten institutions all have more than 40 publications, with Tufts University (0.17) publishing the highest number of papers at 152, ranking first, followed by the University of Pittsburgh (0.07) and the University of Florida (0.07) with 72 and 65, respectively, both being U.S. universities. The countries where the top ten publications are located are mainly concentrated in Europe and North America.

**Table 2:** Distribution of major research institutions (top 10), 2013 to 2022.

	Count	Centrality	Institutions
1	152	0.17	Tufts Univ
2	72	0.07	Univ Pittsburgh
3	65	0.07	Univ Florida
4	52	0.08	Maastricht Univ
5	48	0.05	Univ Melbourne
6	44	0.01	Univ Copenhagen
7	44	0.06	Univ Birmingham
8	43	0.03	Univ Nottingham
9	41	0.01	Univ Sao Paulo
10	41	0.06	Manchester Metropolitan Univ

### 3.4. Author analysis

**Figure 3.**
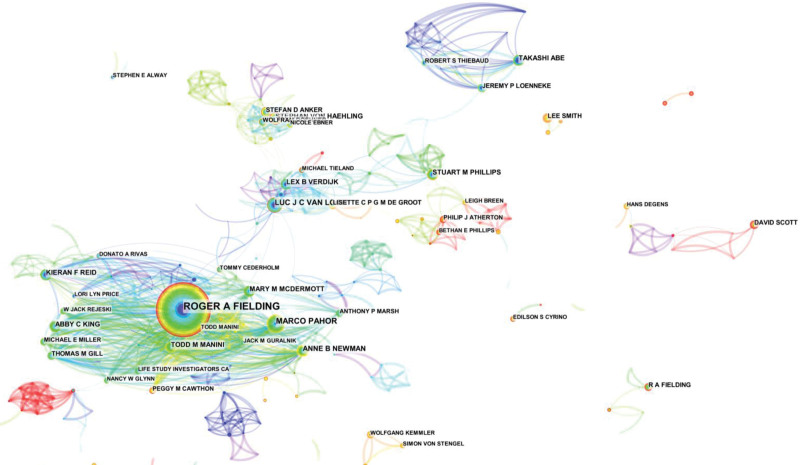
Knowledge map of the distribution of high-yield authors of associated studies, 2013 to 2022.

The authors of the publications were analyzed using CiteSpace to obtain the co-cited authors, as shown in Figure 3 and Table 3. The plot has 883 nodes and 3226 connections, with a density of 0.0083. Larger nodes in the plot indicate more frequent appearances by the authors. The ten authors listed in Table 3 all have more than 20 publications in 2012 to 2022 and are among the most prolific authors in this research area. Among them, ROGER A FIELDING had the highest number of articles (92). His main research areas were nutrition, exercise physiology, and sarcopenia, and the second and third authors were MARCO PAHOR and LUC J C VAN LOON, with 34 and 29 articles, respectively. The Authors with more than 20 publications accounted for 10.36% of all authors publications.

**Table 3:** Distribution of highly prolific authors of related research (top 10), 2013 to 2022.

	Count	Affiliations and countries	Author
1	92	Tufts University (USA)	Roger A Fielding
2	34	University of Florida (USA)	Marco Pahor
3	29	Maastricht University (Netherlands)	Luc J C Van Loon
4	23	University of Pittsburgh (USA)	Anne B Newman
5	22	University Medical Center Göttingen (Germany)	Stephan Von Haehling
6	22	Maastricht University (Netherlands)	Lex B Verdijk
7	20	University of Florida (USA)	Todd M Manini
8	20	Nagahama Institute of Biotechnology (Japan)	Takashi Abe
9	20	McMaster University (Canada)	Stuart M Phillips
10	20	Tufts University (USA)	Kieran F Reid

### 3.5. Keyword analysis

**Figure 4.**
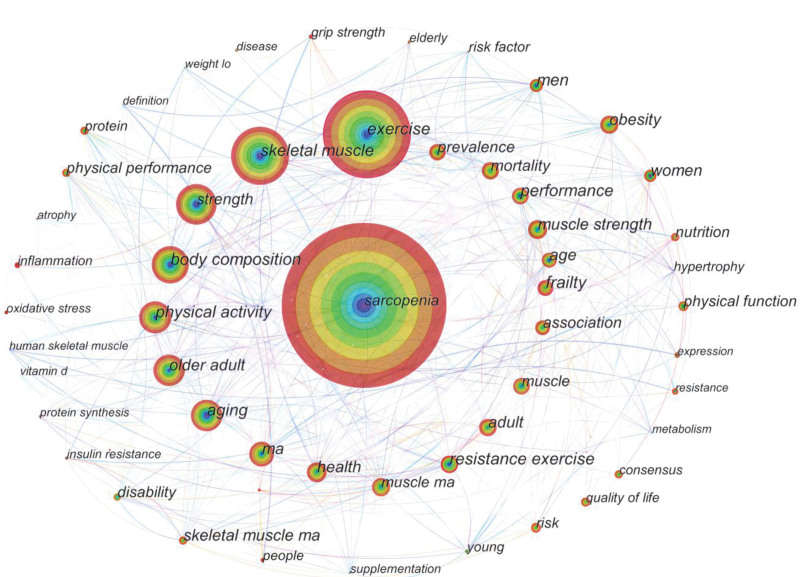
Keyword co-occurrence mapping of exercise therapy for muscle decay syndrome, 2013 to 2022.

**Figure 5.**
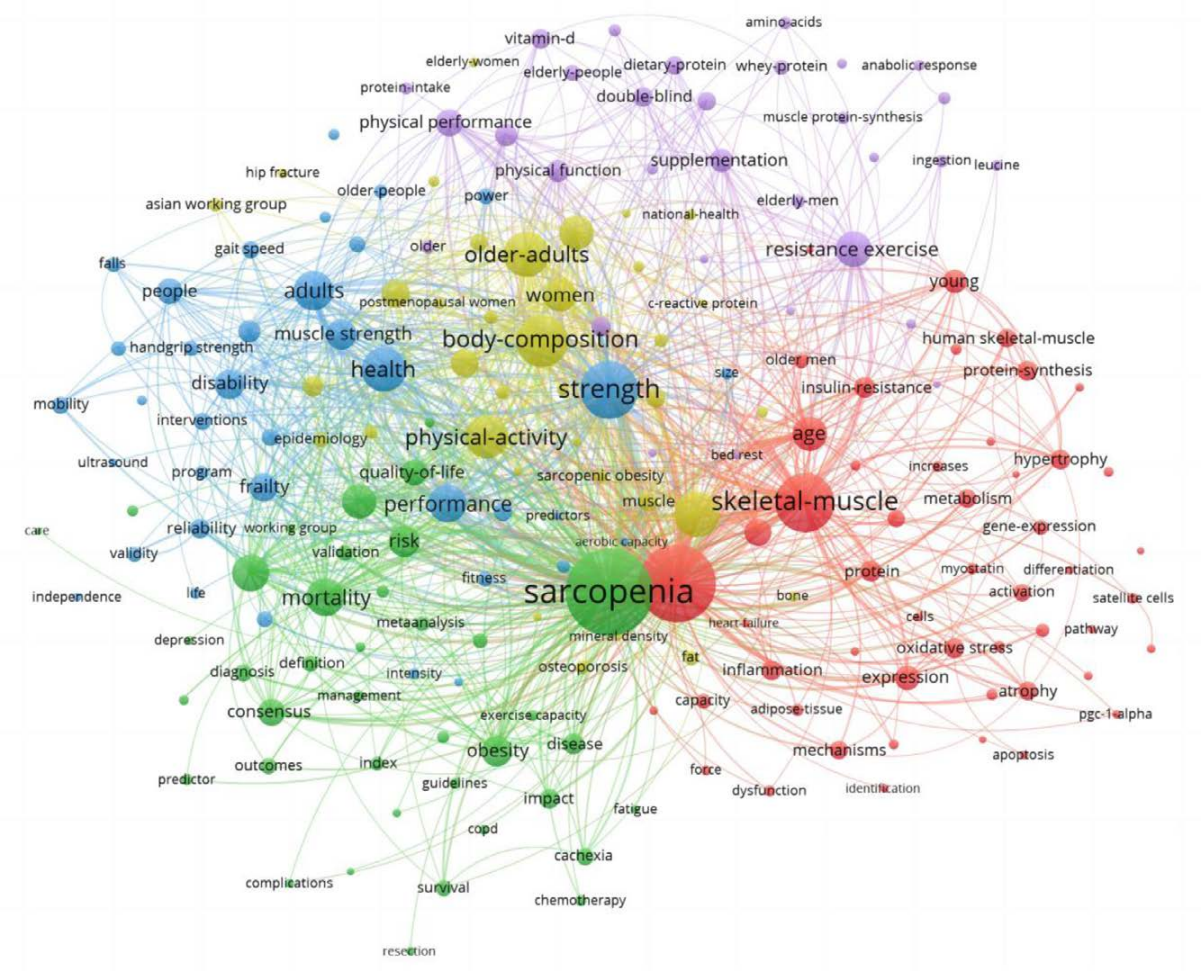
Keyword clustering map for exercise therapy for muscle attenuation syndrome, 2013 to 2022.

Keywords can be used to summarize the focus of the article. In Figure 4, each node represents a keyword. The larger the node, the more frequently a keyword appears, and the thickness of the connecting line represents the strength of the connection between them. CiteSpace shows the relationship between keywords and keywords in the form of pictures by analyzing the information in the article. There are research hotspots in this field. Figure 5 and Table 4 show the top ten appearances were sarcopenia, exercise, skeletal muscle, strength, body composition, older adults, physical activity, aging, mass, and health. Sarcopenia will have the highest number of occurrences in 2044. The occurrences of exercise and skeletal muscle were 1116 and 732, respectively.

**Table 4:** Frequency of keywords for exercise therapy for muscle wasting syndrome, 2013 to 2022.

	Count	Keywords
1	2044	Sarcopenia
2	1116	Exercise
3	732	Skeletal muscle
4	536	Strength
5	494	Body composition
6	439	Older adult
7	432	Physical activity
8	419	Aging
9	340	Ma
10	303	Health ma

The keyword clustering results were mainly in 5 major categories, with 5 colors: green, red, blue, yellow, and purple (see Fig. 5). Green group main keywords: sarcopenia, mortality, consensus, obesity, and disease. Red group main keywords: exercise, skeletal-muscle, oxidative stress, age, and young. Blue group main keywords: Strength, health, adults, disability, frailty, performance. Blue: strength, health, adults, disability, frailty, performance. Yellow: physical activity, body composition, older adults, wrestling. The main keywords in the purple group were resistance exercise, supplementation, and physical performance.

**Figure 6.**
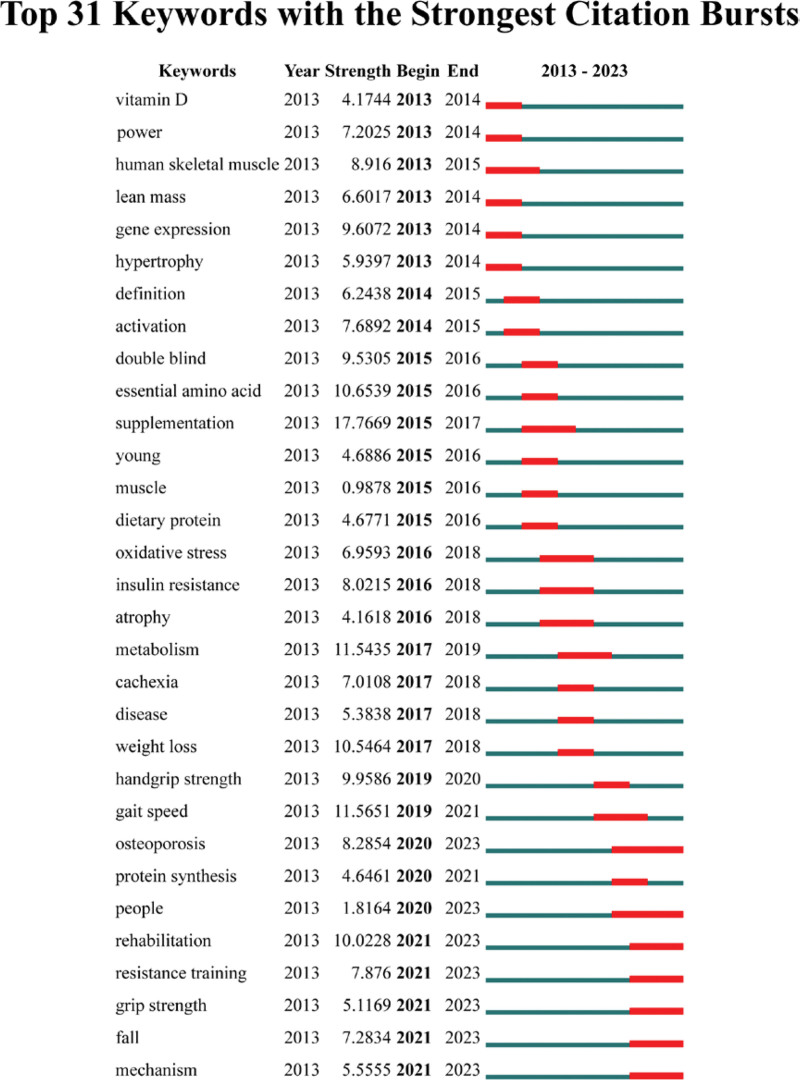
Keyword prominence map for exercise therapy for muscle attenuation syndrome, 2013 to 2022.

The emergence of keywords reflects changes in hotspots and trends in research within the field. We analyzed the included literature, and the results are shown in Figure 6, which displays the top 31 keywords with the strongest emergence. The top 3 keywords with the strongest citation bursts are “supplementation” at 17.7669, “gait speed” at 11.5651, and “metabolism” at 11.5435. Additionally, “rehabilitation,” “resistance training,” “grip strength,” “fall,” and “mechanism” have been more active in the last 3 years.

### 3.6. Highly cited analysis

The co-citation analysis is shown in Table 5, where sarcopenia: revised European consensus on definition and diagnosis has the highest number of citations, 378, published by Cruz-Jentoft in Age Ageing journal in 2019, and this author is also highly cited. It ranked first. The next Asian Working Group for Sarcopenia: 2019 Consensus Update on Sarcopenia Diagnosis and Treatment with 133 citations was Chen, published in J Am Med Dir Assoc. Among the highly cited articles, mainly consensus, only 1 article was a randomized clinical trials.

**Table 5:** Highly cited literature related to muscle attenuation syndrome, 2013 to 2022.

	Count	Periodicals	Yr	Cited references	Thesis title
1	378	Age Ageing	2019	Cruz-Jentoft AJ, 2019, AGE AGEING, V48, P16, DOI 10.1093/aging/afy169	Sarcopenia: revised European consensus on definition and diagnosis
2	133	J Am Med Dir Assoc	2020	Chen LK, 2020, J AM MED DIR ASSOC, V21, P300, DOI 10.1016/j.jamda.2019.12.012	Asian Working Group for Sarcopenia: 2019 Consensus Update on Sarcopenia Diagnosis and Treatment
3	112	J Am Med Dir Assoc	2014	Chen LK, 2014, J AM MED DIR ASSOC, V15, P95, DOI 10.1016/j.jamda.2013.11.025	Sarcopenia in Asia: consensus report of the Asian Working Group for Sarcopenia
4	110	Age Ageing	2010	Cruz-Jentoft AJ, 2010, AGE AGEING, V39, P412, DOI 10.1093/aging/afq034	Sarcopenia: European consensus on definition and diagnosis: Report of the European Working Group on Sarcopenia in Older People
5	88	Age Ageing	2014	Cruz-Jentoft AJ, 2014, AGE AGEING, V43, P748, DOI 10.1093/aging/afu115	Prevalence of and interventions for sarcopenia in aging adults: a systematic review. Report of the International Sarcopenia Initiative (EWGSOP and IWGS)
6	80	Lancet	2019	Cruz-Jentoft AJ, 2019, LANCET, V393, P2636, DOI 10.1016/S0140-6736(19)31138-9	Sarcopenia
7	74	J Am Med Dir Assoc	2011	Fielding RA, 2011, J AM MED DIR ASSOC, V12, P249, DOI 10.1016/j.jamda.2011.01.003	Sarcopenia: an undiagnosed condition in older adults. Current consensus definition: prevalence, etiology, and consequences. International working group on sarcopenia
8	66	J Gerontol A Biol Sci Med Sci	2014	Studenski SA, 2014, J GERONTOL A-BIOL, V69, P547, DOI 10.1093/gerona/glu010	The FNIH sarcopenia project: rationale, study description, conference recommendations, and final estimates
9	63	J Am Med Dir Assoc	2013	Bauer J, 2013, J AM MED DIR ASSOC, V14, P542, DOI 10.1016/j.jamda.2013.05.021	Evidence-based recommendations for optimal dietary protein intake in older people: a position paper from the PROT-AGE Study Group
10	58	JAMA	2014	Pahor M, 2014, JAMA-J AM MED ASSOC, V311, P2387, DOI 10.1001/jama.2014.5616	Effect of structured physical activity on prevention of major mobility disability in older adults: the LIFE study randomized clinical trial

## 4. Discussion

### 4.1. Promotional effect of resistance exercise on muscle reconstruction

Resistance training (RT) mediates the PI3K/Akt/mTOR pathway^[12,13]^ and autophagy pathway of mTOR/ULK1. It also elevates muscle oxygen consumption, increases capillary muscle density,^[14]^ promotes muscle protein synthesis, mediates the Beclin1-Vps pathway, inhibits phosphorylation of FoxO3, downregulates autophagy levels, reduces protein degradation in muscle, and stabilizes skeletal muscle mass stable.^[15]^ Therefore, resistance exercise is the best way to enhance muscle strength and mass in the elderly, which can effectively prevent the decline in physical function and disability caused by muscle decay and improve the quality of life. A deeper analysis of the highly cited literature shows that progressive resistance exercise and blood flow restriction training (BFRT) methods are among the hot research topics in exercise therapy for sarcopenia.

#### 4.1.1. Mitigating effect of progressive resistance training (PRT) on muscle aging.

PRT refers to the production of continuous adaptive stimulation of the muscles, thus improving strength by increasing the resistance load during training. According to the American College of Sports Medicine guidelines on exercise testing and prescription, people who have previously participated in similar activities can adapt better to high-intensity exercise. For those without such an experience, moderate-intensity exercise is a more appropriate choice. In addition, older adults limited to low-intensity training are best served by gradually increasing the number and length of sessions to achieve better health outcomes.^[16]^ Davis used an isokinetic dynamometer (Biodex System 3) and a pneumatic bilateral seated leg press (K400, Keizer Sports Health Equipment, Fresno, CA) to examine the effects of 12 weeks of PRT (two sets of 10 repetitions progressing over 12 weeks to 3 sets of 12 repetitions at 80% 1RM) on knee flexion in immobile older adults (age 70–92 years). They found that the training significantly improved the isometric and isotonic peak torque of the knee flexors and extensors and leg press strength and power.^[17]^

The ability of PRT to increase not only muscle strength but also torque was confirmed. A meta-analysis of 121 studies on the effects of progressive resistance exercise on physical function in older adults by Liu et al found that PRT performed 2 to 3 times per week improved physical function in older adults. The improvement included reduced physical disability; some functional limitations (i.e., balance; gait speed; timed walking; timed “get up and go”; chair rise; and stair climbing); and muscle weakness.^[18]^ Steven meta-analysis found that 3 PRT per week can maximize muscle and bone strength. These exercises include weight/impact loading exercises (e.g., jumps, strides) and require 1 or 2 sets per exercise with a load equivalent to 75% to 80% for a maximum of 1 repetition.

Single progressive resistance exercises are being validated to promote physical fitness in older adults. Researchers are also beginning to focus on using advanced resistance exercises in combination with other movements and comparing the effects of different forms of PRT. Jennifer et al combined progressive resistance exercise and lower extremity balance training in long-term hospitalized older adults (65–100 years) for a 25-week intervention to reduce fall rates and improve physical function in elderly care residents.^[19]^ Progressive resistance exercises with fast-speed strength exercises can bridge the deficit of low loads. Dennis et al studied high-and low-speed progressive resistance exercises (5–15 repetitions) for 14 weeks. They found that RT with faster speed and lower load was as practical as slower speed and higher load in stimulating type 2 muscle fibers.^[20]^ Theraband elastic resistance bands are a new approach to RT, with different colors representing different. They are gradually being used in rehabilitation programmes. Patil recent study used Thera-Band elastic bands for 6 weeks in stroke patients and positively improved gait quality and functional mobility.^[21]^

#### 4.1.2. BFRT can safely slow muscle aging.

BFRT, also known as KAATSU training, is a RT method that blocks the restriction of local blood flow to promote protein synthesis, stimulate muscle growth, and improve muscle fitness.^[22]^ A meta-analysis showed that a weekly set of high-intensity (70%–85% 1RM2-3/reps) RT with 6 to 12 repetitions for 8 to 12 weeks was the minimum effective dose to increase 1RM strength.^[23]^ However, recent studies have shown that BFRT combined with low-load RT produces similar muscle hypertrophy and strength responses to those following high-load intensity training. Christopher Low-intensity BFRT was performed in elderly subjects (70 ± 2 years) with bilateral leg extensions at 20% of the maximum number of repetitions (1-RM) and a pressure cuff applied at 200 mm Hg. mTORC1 downstream, ribosomal S6 kinase 1 (S6K1) phosphorylation and ribosomal protein S6 (rpS6) phosphorylation were significantly increased. Low-intensity BFRT enhances mTORC1 signaling and muscle protein synthesis in older men, and is a novel rehabilitation intervention for muscle wasting syndrome.^[24]^

Owing to the intervention characteristics, the cuff exerts pressure on the participant during BFR exercise, and safety has caused researchers to think about it. One study confirmed that BFRT increases muscle function in older adults without negatively affecting their safety. Nakajima found no significant increase in intravascular clot formation, markers of fibrin degradation product D-dimer and fibrin degradation products, or markers of coagulation activity, prothrombin time and thrombin time after low-intensity BFRT.^[25]^ Madarame et al demonstrated that in cardiac patients 4 sets of low-intensity RT-BFR did not increase fibrinogen/fibrin degradation products and high-sensitivity C-reactive protein levels, suggesting that BFRT does not affect hemostasis and inflammatory responses.^[26]^

### 4.2. Aerobic exercise (AE) alleviates muscle aging and improves athletic performance

#### 4.2.1. AE increases myosin heavy chain plasticity.

The adaptive changes in AE are mainly reflected in the increase in mitochondrial number, change in myosin heavy chain (MHC) from fast to slow, and overall enhancement of muscle function.^[27]^ Naito endurance training of specific pathogen-free male rats revealed that AE increased α-actinin-2 expression in skeletal muscle.^[28]^ Adam et al found that aerobic training for 12 weeks in older women decreased MHCIIa and IIx levels of mRNA and protein. The increase in aerobic training-induced MHCI protein and mRNA levels suggests that skeletal muscle plasticity is maintained with age.^[29]^ Prior studies found that training increased skeletal muscle capillary density in older adults through 6 months of AE, and that the increase in skeletal muscle capillary density contributed to a sustained improvement in glucose metabolism, thus contributing to regular physiological activity of the body.^[30]^

#### 4.2.2. Promotional effects of aerobic combined resistance exercise on muscle function in the elderly.

Both aerobic and resistance exercises alone can help prevent, slow, and improve muscle wasting syndromes. However, muscle mass and functional improvements vary more in older adults. Several studies have demonstrated that aerobics, combined with resistance and other forms of exercise, are more effective in promoting physical fitness in older adults. A meta-analysis showed that older adults with sarcopenia who performed AE combined with RT and whole-body vibration training increased their knee extension strength more than those who did not, with no significant difference between the latter group and the controls.^[31]^ The researchers concluded that although both AE combined with RT and whole-body vibration training can improve muscle aging in older adults, the former has a greater advantage in improving overall muscle function. Aerobic training combined with RT programs are often scheduled in 1 training session, that is, concurrent training. Most studies on the interference effects of concurrent training have focused on the development of strength and explosive power, and few have explored and analyzed skeletal muscle hypertrophy as a variable. Osuka et al believed that concurrent training negatively affected skeletal muscle hypertrophy.^[32,33]^ However, Mikkola et al (2014) concluded that concurrent training did not disrupt skeletal muscle hypertrophy.^[34]^ There is no consensus in the academic community regarding the interference effect of concurrent training on skeletal muscle hypertrophy. The differences in the effects of concurrent training on skeletal muscle hypertrophy may be due to the different experimental designs of the studies. Several factors influence the effects of muscle hypertrophy after training. For example, differences in training duration, subjects’ training background, age, and gender, as well as training mode, frequency, and intensity exert different stimuli on the organism. These stimuli affect the degree of molecular signal transduction and protein synthesis in the muscle after training, and ultimately have different effects on muscle hypertrophy.

Therefore, future studies should fully consider the effects of experimental variables on the final muscle hypertrophy effect when conducting experimental designs on the results of concurrent training on skeletal muscle hypertrophy, and should also conduct in-depth studies from the perspective of molecular biology.

### 4.3. Exercise therapy combined with nutritional supplementation to improve muscle decay

Current research on the nutritional treatment of sarcopenia has focused on protein, creatine, amino acids, vitamin D, lipids, and proinflammatory diet supplementation.^[35]^ Protein and energy malnutrition increases the risk of sarcopenia in older adults. A proper diet is also crucial for promoting muscle protein synthesis to improve muscle mass in patients with muscle wasting syndrome. Studies have shown that Exercise therapy combined with nutritional supplementation is the best option for maintaining muscle function and preventing muscle wasting.^[36]^

#### 4.3.1. Exercise combined with protein supplementation has an important role.

Studies have shown that exercise and nutrition improve muscle mass, strength, and physical function compared to no intervention.^[37,38]^ Xie et al found that the combination of training and nutrition significantly helped frail or frail older adults improve their frailty scores and physical performance through exercise and nutrition interventions for frail older adults. Similarly, there is evidence of the benefits of the combined intervention regarding walking parameters, flexibility, level of physical activity, and activities of daily living.^[37–39]^ Both randomized controlled trials that applied RT only showed the benefits of combining exercise and nutrition. In contrast, the benefits were inconsistent in studies that included aerobic, balance, flexibility, and gait training. Regarding methodology, evidence of benefits was consistent in individualized program studies.^[39,40]^ Thus, there is reasonable evidence that the addition of an exercise component and other dietary/nutritional interventions in selected subjects would provide additional benefits.

#### 4.3.2. Exercise combined with creatine supplementation is the most widely used.

Creatine has been one of the most common sports supplements used by athletes since the 1990s, and is especially important in fitness and bodybuilding sports groups. It is a cellular energy buffer that enhances the regeneration of phosphocreatine and adenosine triphosphate. It also increases hydration and accelerates the rate of protein synthesis, leading to improvements in muscle size, strength, and endurance.^[41]^ Exercise-induced muscle damage had been found to lead to adverse effects such as increased soreness, impaired muscle function, and decreased muscle strength. Creatine supplementation has a positive effect on the recovery of delayed muscle soreness. The supplementation group had significantly lower levels of creatine kinase and lactate dehydrogenase than the placebo group, indicating that creatine supplementation can reduce muscle damage after exercise and promote body recovery.^[42]^ Arazi et al reviewed the literature on creatine and found that it has multiple benefits for the body. Creatine increases serum C-reactive protein reserves and improves type II muscle fibers. However, it also protects mtDNA and RNA from oxidative damage and enhances the antioxidant system when combined with long-term training. Most studies have demonstrated the positive benefits of creatine and exercise, and its safety and tolerability have been confirmed in clinical studies.^[43]^

#### 4.3.3. Other nutritional supplements.

Amino acids can improve athletic performance by altering energy utilization during exercise and by preventing neurological fatigue and overtraining. In 2017, a statement published by the International Society of Sports Nutrition noted that branched-chain amino acid (BCAA) supplementation is essential among all essential amino acids because of its unique role in protein metabolism, neurological function, and blood glucose and insulin regulation.^[44]^ A summary of the literature on BCAAs by Khemtong et al found that BCAA supplementation after resistance exercise showed a positive effect on reducing CK concentrations and reducing muscle soreness, but not LDH concentrations when the study population was only men.^[45]^ These results suggests that BCAA supplementation does not prevent muscle damage but promotes the regression of inflammation by activating cell regeneration.

Vitamin D triggers genomic and non-genomic pathways in muscle cells that maintain muscle function through various mechanisms, including the maintenance of calcium homeostasis and muscle fiber proliferation. Multiple cross-sectional studies in Asia (N = 11769) provide indirect evidence for the role of vitamin D supplementation in muscle health.^[46]^

While protein is a component of muscle, lipids, such as omega-3 or n-3 polyunsaturated fatty acids, are thought to indirectly affect muscle health by preventing low levels of inflammation. Significantly lower serum n-3 fatty acid levels are associated with sarcopenia, and a higher dietary fat intake is associated with lower odds of sarcopenia.

Diets with pro-inflammatory potentials are associated with a higher risk of sarcopenia.^[47–49]^ Therefore, diets with anti-inflammatory potential, that is, diets that include foods rich in specific antioxidants, may prevent this condition. In a cross-sectional study (n = 2451), dietary and bell pepper (rich in antioxidants) consumption were assessed using a self-administered food frequency questionnaire.^[50]^ Increased consumption of peppers and bell peppers is significantly associated with a reduced risk of sarcopenia. This suggests that capsaicin and capsaicinoids (active anti-inflammatory compounds in these foods) may help prevent sarcopenic episodes.

## 5. Conclusion

This article presents the first visual analysis of literature records on exercise therapy for sarcopenia in older adults included in the Web of Science core set database over the past ten years. Exercise intervention is an important and continuously developing research direction for sarcopenia in older adults. Myasthenia gravis in the elderly is a complex disease that involves multiple factors. Research on the efficacy of exercise for this disease in the last decade has focused on 3 aspects of exercise therapy for myasthenia gravis, RT, RT combined with other types of exercise, and training combined with nutritional supplementation. The research content has positive guidance for promoting myasthenia gravis in the elderly. Sarcopenia in the elderly is associated with the risk of debilitating type 2 diabetes, heart failure, and other diseases. It also affects the prognosis of certain diseases. Therefore, building new models and strategies for exercise-nutrition interventions for sarcopenia under different conditions will become a hot research direction in the future.

## Author contributions

**Methodology:** Wanli Zang.

**Project administration:** Wanli Zang.

**Resources:** Wanli Zang.

**Software:** Wanli Zang, Haohao Chen, Jin Yan.

**Supervision:** Wanli Zang.

**Validation:** Wanli Zang, Zezhong Zhang.

**Visualization:** Wanli Zang, Zezhong Zhang.

**Writing – original draft:** Wanli Zang, Jin Yan, Dong Li, Zezhong Zhang.

**Writing – review & editing:** Wanli Zang, Haohao Chen, Dong Li, Ningkun Xiao, Xiaoqin Zheng, Zezhong Zhang.
